# Using In-Shoe Inertial Measurement Unit Sensors to Understand Daily-Life Gait Characteristics in Patients With Distal Radius Fractures During 6 Months of Recovery: Cross-Sectional Study

**DOI:** 10.2196/55178

**Published:** 2024-03-20

**Authors:** Akiko Yamamoto, Eriku Yamada, Takuya Ibara, Fumiyuki Nihey, Takuma Inai, Kazuya Tsukamoto, Tomohiko Waki, Toshitaka Yoshii, Yoshiyuki Kobayashi, Kentaro Nakahara, Koji Fujita

**Affiliations:** 1 Department of Orthopaedic and Spinal Surgery Graduate School of Medical and Dental Sciences Tokyo Medical and Dental University Tokyo Japan; 2 Department of Functional Joint Anatomy Graduate School of Medical and Dental Sciences Tokyo Medical and Dental University Tokyo Japan; 3 Biometrics Research Laboratories NEC Corporation Chiba Japan; 4 Biomechanics and Exercise Physiology Research Group Health and Medical Research Institute, Department of Life Science and Technology National Institute of Advanced Industrial Science and Technology Kagawa Japan; 5 Human Augmentation Research Center National Institute of Advanced Industrial Science and Technology Tokyo Japan; 6 Division of Medical Design Innovations Open Innovation Center, Institute of Research Innovation Tokyo Medical and Dental University Tokyo Japan

**Keywords:** distal radius fracture, gait analysis, daily life, long-term results, gait, sensor, sensors, walk, walking, fracture, fractures, wearable, wearables, recover, rehabilitation, spatiotemporal, inertial measurement, fragility, postmenopausal, menopause, grip, surgery, surgical, orthopedic, postoperative, orthopedics, fall, falls, bone, bones, wrist, radius, radial

## Abstract

**Background:**

A distal radius fracture (DRF) is a common initial fragility fracture among women in their early postmenopausal period, which is associated with an increased risk of subsequent fractures. Gait assessments are valuable for evaluating fracture risk; inertial measurement units (IMUs) have been widely used to assess gait under free-living conditions. However, little is known about long-term changes in patients with DRF, especially concerning daily-life gait. We hypothesized that, in the long term, the daily-life gait parameters in patients with DRF could enable us to reveal future risk factors for falls and fractures.

**Objective:**

This study assessed the spatiotemporal characteristics of patients with DRF at 4 weeks and 6 months of recovery.

**Methods:**

We recruited 16 women in their postmenopausal period with DRF as their first fragility fracture (mean age 62.3, SD 7.0 years) and 28 matched healthy controls (mean age 65.6, SD 8.0 years). Daily-life gait assessments and physical assessments, such as hand grip strength (HGS), were performed using an in-shoe IMU sensor. Participants’ results were compared with those of the control group, and their recovery was assessed for 6 months after the fracture.

**Results:**

In the fracture group, at 4 weeks after DRF, lower foot height in the swing phase (*P*=.049) and higher variability of stride length (*P*=.03) were observed, which improved gradually. However, the dorsiflexion angle in the fracture group tended to be lower consistently during 6 months (at 4 weeks: *P*=.06; during 6 months: *P*=.07). As for the physical assessments, the fracture group showed lower HGS at all time points (at 4 weeks: *P*<.001; during 6 months: *P*=.04), despite significant improvement at 6 months (*P*<.001).

**Conclusions:**

With an in-shoe IMU sensor, we discovered the recovery of spatiotemporal gait characteristics 6 months after DRF surgery without the participants’ awareness. The consistently unchanged dorsiflexion angle in the swing phase and lower HGS could be associated with fracture risk, implying the high clinical importance of appropriate interventions for patients with DRF to prevent future fractures. These results could be applied to a screening tool for evaluating the risk of falls and fractures, which may contribute to constructing a new health care system using wearable devices in the near future.

## Introduction

Gait analysis is useful for predicting future fall risk and reflecting various underlying physiological processes [1]. Quantitative gait characteristics, such as slower gait speed and shorter stride length, are associated with falls, resulting in fragility fractures [2-4]. Recently, inertial measurement units (IMUs) have been widely used to assess gait under free-living conditions owing to their convenience, low cost, small size, and high accuracy [5-7]. The shank and foot are the preferred placements [8,9], and foot kinematics is an important factor related to falls and physical ability [4].

Among fragility fractures resulting from falls, distal radius fractures (DRFs) are one of the most frequent initial fractures in older adults [10]. Many DRFs occur in women in their postmenopausal period, aged <75 years, who are healthy, active, and functionally independent. More than half of these women do not meet the criteria for osteoporosis [11-13]. However, the initial DRF is associated with a greater risk of functional decline [14] and subsequent fractures [15] in all age groups. These changes are more than 5 times higher, even in those aged 50-59 years [16]. This could reflect early changes in frailty [17].

In patients with DRF, lower gait ability was observed in the laboratory, which slightly improved 6 months after the surgery [13,18]. However, these gait assessments were mainly performed for approximately 10 seconds, which may not accurately depict daily-life gait [19]. Further, most studies have only highlighted the consequences of wrist function and pain when investigating the long-term outcomes of DRF, and little is known about the effect of DRF on physical abilities, such as activities of daily living. Therefore, the long-term alterations of daily-life gait characteristics in patients with fractures remain unknown, and this study attempts to bridge this gap.

We previously found out that in-shoe IMU sensors were effective in the assessment of daily-life gait in patients with an initial DRF [7]. We hypothesized that, in the long term, it could enable us to identify future risk factors for secondary fractures by spatiotemporally following daily-life gait parameters using IMU sensors. We aimed to reveal the characteristics of spatiotemporal gait changes during 6 months following DRF.

## Methods

### Ethical Considerations

This study was approved by the Institutional Review Board of Tokyo Medical and Dental University (M2020-365) and followed the tenets of the Declaration of Helsinki. Written informed consent was provided by all participants. Participation in the study was voluntary, and no compensation was awarded for participation.

### Recruitment

In the fracture group, we recruited 16 female patients with DRF who had undergone surgery for their first fragility fracture from 5 general hospitals. We compared their results to those of 28 healthy female volunteers. Women without a history of fragility fractures were recruited as the control group through local media advertisements. The inclusion criteria involved having the ability to walk without any support, no history of lower-extremity injury, and no known neuromuscular disorders or neurophysiological problems that may affect gait. Fragility fractures were defined as those that followed a fall from standing height or less. We excluded patients with DRF due to traffic or industrial accidents or multiorgan injuries. In the fracture group, 6 patients with DRF fell in the house without shoes, and the other 10 patients fell while wearing shoes. Owing to the lack of previous literature on the long-term data of gait in patients with DRF, the sample size estimations were based on the effect size of 0.78 (from the result of hand grip strength [HGS]) [13], with an assumed power of 0.8 and a type I error of 0.05. A sample of 16 participants with fractures was analyzed using G*Power (version 3.1; Heinrich Heine University Duesseldorf) [20].

### Daily-Life Gait Assessments

**Figure 1:**
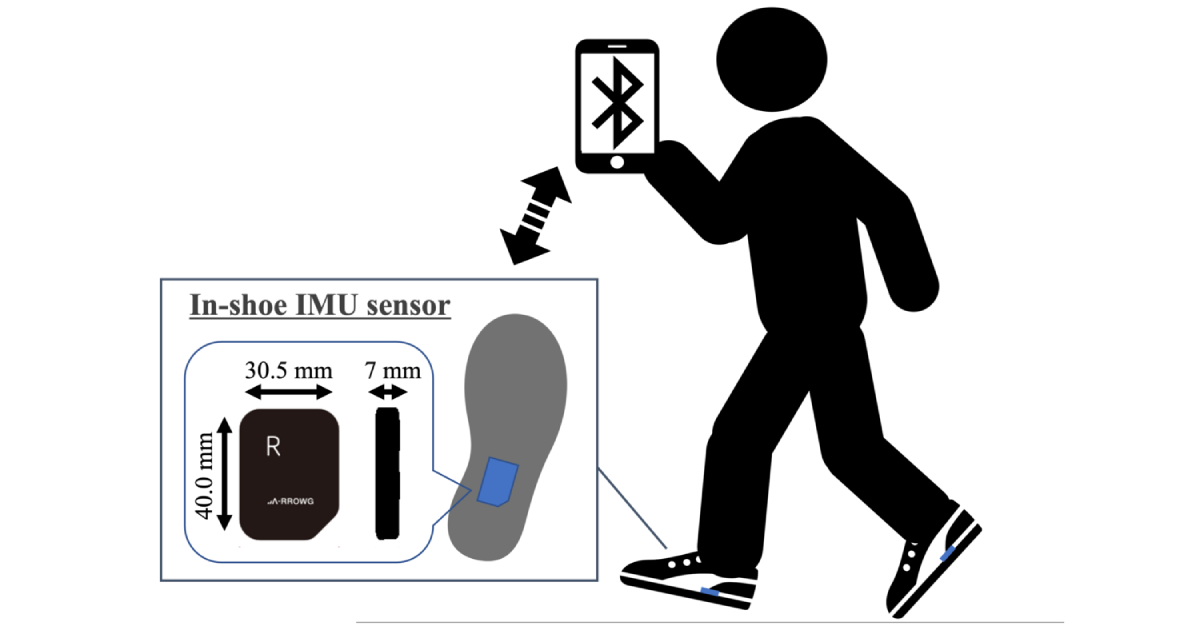
The in-shoe inertial measurement unit (IMU) sensor. The sensors with accelerometer and gyroscope were set into the dedicated insoles, measured the daily-life gait without the participants’ awareness, and saved the obtained data to their smartphone via Bluetooth.

We measured daily-life gait using in-shoe IMU sensors (A-RROWG, NEC Corporation; Figure 1). These sensors are small (40.0 mm × 30.5 mm × 7 mm) and lightweight (11 g), including a 3-axis accelerometer and gyroscope. The IMU sensor in the dedicated insole was placed at the foot arch, and the x-, y-, and z-axes of the IMUs were set along the mediolateral, anteroposterior, and vertical directions, respectively. When a person wearing these sensors walks in a stable straight line over 3 gait cycles between 5 AM and 10 PM, the in-shoe IMU sensor detects that the person is walking based on acceleration in the anteroposterior direction and saves the IMU signals of the next 3 gait cycles as 1 gait measurement [21]. The IMU signals were sampled at a rate of 100 Hz, transferred to a smartphone via Bluetooth, and stored in a specialized app if participants had one with them. If a person did not have a smartphone, the data were uploaded automatically via Bluetooth at 11 PM by keeping the smartphone near the IMU sensors.

From the saved IMU signals, the mean of 7 gait parameters from 3 gait cycles was calculated and stored on a smartphone, as previously described by Fukushi et al [21]. The following 7 parameters were calculated:

1. Gait speed: calculated as stride length (m) divided by stride time (s).
2. Stride length: the distance between the start and end points of the foot trajectory for 1 stride.
3. Dorsiflexion angle: the peak foot angle in the dorsal direction from the ground during the swing phase.
4. Plantarflexion angle: the peak foot angle in the plantar direction from the ground during the swing phase.
5. Foot height: the maximum height of the foot trajectory.
6. Toe-in or toe-out angle: the mean angle of foot adduction or abduction in the direction of the velocity vector during the swing phase.
7. Circumduction: the displacement in the medial-lateral direction during the swing phase.

In addition to these 7 gait parameters, the coefficient of variation (CV), calculated as SD divided by mean multiplied by 100, was used to evaluate the variability.

### Measurement Protocol

All participants completed a paper-based questionnaire on their general health status, which included fall history in the past year, frequency of stumbling, and fear of falling. Falls at the time of fracture in patients with DRFs were excluded based on the number of falls in the past year.

In the daily gait assessments, we inserted IMU sensors with dedicated insoles into the preferred shoes of both participants’ feet. Each participant was provided a smartphone with only the original app for storing gait data. We verified whether participants could walk using the sensors and whether the sensors worked with a smartphone. Participants were instructed to wear shoes with sensors for 2 to 6 weeks. We requested that participants spend their daily lives as usual; therefore, we did not establish a minimum time for wearing or walking with them. The measurements were recorded without the participants’ awareness, and they could view their most recent gait data by checking their smartphone. In the fracture group, daily gait assessments in the early postoperative period started 2 weeks after DRF surgery to enable the effects of casting or surgery.

To assess baseline functional ability and frailty, HGS [22] and the Timed Up and Go (TUG) test [23] were performed. HGS was measured in kilograms with a Jamer dynamometer (Sammons Preston). We assessed HGS on the nonfractured side of the fracture group and both sides of the control group. The mean values of 3 measurements were recorded. The time to complete the TUG test was recorded twice: once at the preferred speed and once at the fastest speed. These baseline physical tests were performed 4 weeks after DRF surgery in the fracture group and at the beginning of daily gait measurements in the control group.

To check for long-term functional ability, we asked the fracture group patients to perform the same physical and daily gait assessments again at an outpatient visit 6 months after the surgery.

### Data Analysis

The median and CV of each participant were calculated using the obtained gait data. However, the automatically collected data included hills, turns, and straddling, and we used Smirnov-Grubbs analysis for every gait parameter to exclude any outliers from the data. After exclusion, approximately 20-1000 gait measurements were recorded for each participant during the measurement period, reflecting differences in their lifestyles. Even after the exclusion, gait data included various gait types, such as walking exercise and hurried walking; therefore, we excluded participants with less than 50 gait measurements in either foot, based on a previous report [24]. Since we computed the data obtained from both feet together, we selected participants with 100 or more gait assessments for this study.

### Statistical Analysis

Between-group differences were assessed using Student *t* test (2-tailed) for continuous variables in the patient demographics, and the chi-square test for categorical variables. Since the participants’ gait data were not normally distributed, they were assessed using nonparametric (ie, Kruskal-Wallis and Steel) tests. The results of the fracture group in the early postoperative and long-term periods were compared using a paired *t* test. Statistical significance was set at *P*<.05.

These analyses were performed using EZR (version 1.55; Division of Haematology, Saitama Medical Center, Jichi Medical University) [25].

## Results

### Participants’ Demographics

There were no significant differences in age, body characteristics, or lifestyle variables between the 2 groups. In the section on questions about falls, patients with fractures demonstrated a significantly higher incidence of falls (Table 1). None of the fracture group members experienced falls or subsequent fractures after the initial DRF.

**Table 1:** Participants’ demographics. *P* values <.05 are considered significant.

Characteristics			Control (n=28)		Fracture (n=16)		*P* value
Age (years), mean (SD)			62.3 (7.0)		65.6 (8.0)		.20^a^
Height (cm), mean (SD)			155.2 (4.3)		154.8 (4.0)		.77^a^
Body weight (kg), mean (SD)			54.4 (8.0)		51.6 (8.5)		.30^a^
BMI (kg/m^2^), mean (SD)			22.6 (3.2)		21.5 (3.2)		.28^a^
Hand dominance (right), n (%)			27 (96)		15 (94)		.68^b^
Foot dominance (right), n (%)			23 (82)		14 (88)		.64^b^
Smoking (current and previous), n (%)			5 (18)		5 (31)		.31^b^
Alcohol consumption, n (%)			11 (39)		5 (31)		.59^b^
**Comorbidities, n (%)**							
	Hypertension	8 (29)		3 (19)		.47^b^	
	Eye disease	1 (4)		1 (6)		.68^b^	
	Diabetes mellitus	0 (0)		0 (0)		.48^b^	
	Knee osteoarthritis	2 (7)		0 (0)		.27^b^	
	Hip osteoarthritis	0 (0)		0 (0)		.48^b^	
	Rheumatoid arthritis	0 (0)		0 (0)		.48^b^	
Number of oral medications, mean (SD)			0.8 (0.03)		0.9 (0.06)		.27^a^
The experience of fall in the past year, n (%)			0 (0)		4 (25)		.006^b^
**Number of falls, n**							
	Once	N/A^c^		1		N/A	
	twice	N/A		2		N/A	
	3 times	N/A		1		N/A	
The experience of stumbling, n (%)			17 (61)		9 (56)		.77^b^

^a^Independent Student *t* tests were used to compare the groups.

^b^Chi-square test was used for analysis between the groups.

^c^N/A: not applicable.

### Spatiotemporal Data in Daily-Life Gait

The results of the spatiotemporal daily-life gait are presented in Table 2. There were no between-group differences in the number of measurements. Compared with the control group, the dorsiflexion angle measured at any time postoperatively in the fracture group tended to be lower and demonstrated no improvement in the postoperative course. Participants in the fracture group demonstrated significant recovery in gait speed, stride length, and plantarflexion angle between 4 weeks and 6 months postoperatively. Among the CV of each gait parameter, only the CV of stride length between the control group and the 4 weeks–postfracture group demonstrated a significant difference.

**Table 2:** Daily-life spatiotemporal data. *P* values <.05 are considered significant.

Characteristics			Control group (n=28)		Fracture group (n=16)					*P* value						
					4 weeks after surgery		6 months after surgery		Kruskal-Wallis test			Control (at 4 weeks)		Control (at 6 months)		Control (4 weeks to 6 months)
Number of measurements, mean (SD)			479.3 (432.7)		746.6 (468.7)		543.3 (475.8)		.06^a^			.05^b^		.97^b^		.06^c^
**Median of each parameter, mean (SD)**																
	Gait speed (m/s)	1.28 (0.12)		1.22 (0.09)		1.26 (0.10)		.17^a^			.12^b^		.66^b^		.046^c^	
	Stride length (m)	1.26 (0.12)		1.20 (0.09)		1.24 (0.09)		.26^a^			.23^b^		.94^b^		.001^c^	
	Dorsiflexion angle (degree)	26.1 (3.83)		22.8 (4.15)		23.1 (3.46)		.03^a^			.06^b^		.07^b^		.24^c^	
	Plantarflexion angle (degree)	75.0 (6.18)		71.5 (4.23)		72.8 (4.64)		.08^a^			.06^b^		.31^b^		.04^c^	
	Foot height (cm)	14.0 (1.06)		13.1 (1.35)		13.8 (1.38)		.08^a^			.049^b^		.87^b^		.10^c^	
	Circumduction (cm)	2.85 (0.85)		3.16 (0.49)		3.17 (0.85)		.12^a^			.09^b^		.30^b^		>.99^c^	
	Toe-in or toe-out angle (degree)	13.2 (4.63)		13.6 (3.90)		14.1 (3.97)		.93^a^			>.99^b^		.89^b^		.41^c^	
**CV^d^ of each parameter (%), mean (SD)**																
	Gait speed	15.2 (4.84)		16.3 (2.95)		15.8 (2.96)		.36^a^			.37^b^		.52^b^		.45^c^	
	Stride length	10.3 (2.89)		12.6 (3.21)		11.2 (2.60)		.05^a^			.03^b^		.57^b^		.15^c^	
	Dorsiflexion angle	20.7 (6.09)		23.2 (5.48)		22.9 (4.06)		.19^a^			.22^b^		.31^b^		.74^c^	
	Plantarflexion angle	8.56 (2.74)		10.2 (2.70)		10.1 (3.57)		.14^a^			.12^b^		.34^b^		.65^c^	
	Foot height	8.01 (2.15)		11.0 (7.05)		9.05 (2.73)		.12^a^			.10^b^		.44^b^		.33^c^	
	Circumduction	51.3 (12.8)		46.5 (12.0)		46.5 (6.16)		.45^a^			.79^b^		.39^b^		.83^c^	
	Toe-in or toe-out angle	30.7 (15.3)		37.9 (19.9)		31.7 (13.0)		.51^a^			.44^b^		.89^b^		.15^c^	

^a^Kruskal-Wallis test was used to compare the control and fracture groups.

^b^Steel test was used to compare each group.

^c^Paired sample *t* test was used for analysis between the groups.

^d^CV: coefficient of variation.

### HGS and Body Balancing Ability

The HGS in the fracture group demonstrated significant recovery between 4 weeks and 6 months after surgery; however, it was significantly lower in the fracture group compared to the control group. In the TUG test, there were no significant differences between the control and fracture groups or at 4 weeks and 6 months postoperatively in the fracture group (Table 3).

**Table 3:** Physical tests in the control and fracture groups. *P* values <.05 are considered significant.

Characteristics			Control group (n=28)		Fracture group (n=16)					*P* value						
					4 weeks after surgery		6 months after surgery		Kruskal-Wallis test			Control (at 4 weeks)		Control (at 6 months)		Control (4 weeks to 6 months)
Hand grip strength (kg), mean (SD)			23.3 (3.4)		19.1 (2.6)		20.6 (3.1)		<.001^a^			<.001^b^		.04^b^		<.001^c^
**TUG^d^ test (s), mean (SD)**																
	Normal speed	8.07 (1.33)		7.53 (0.85)		8.2 (1.28)		.28^a^			.29^b^		.96^b^		.13^c^	
	Faster speed	6.23 (0.89)		6.09 (0.64)		6.4 (0.95)		.48^a^			.51^b^		.99^b^		.47^c^	

^a^Kruskal-Wallis test was used to compare the control and fracture groups.

^b^Steel test was used to compare each group.

^c^Paired sample *t* test was used for analysis between the groups.

^d^TUG: Timed Up and Go.

## Discussion

### Principal Results

We performed daily-life gait assessments with in-shoe IMU sensors and some physical tests, including HGS, to evaluate the differences in spatiotemporal gait and physical ability between patients with DRF and healthy controls. Moreover, we assessed whether these parameters improved during 6 months following DRF. In daily-life gait assessments, patients with DRF demonstrated a lower foot height and higher CV stride length compared to the control group; however, these differences were no longer present 6 months after DRF surgery, reflecting the improvement in some parameters in the fracture group after 6 months of DRF treatment. On the other hand, the dorsiflexion angle in the fracture group tended to be lower consistently during 6 months. In the physical assessments, patients with DRF at 4 weeks and 6 months after the surgery had significantly lower HGS than those without DRF, even though HGS in patients with DRF improved during 6 months.

Some gait parameters, such as gait speed and stride length, improved in the fracture group during 6 months of daily life. Further, the CV of stride length, which is associated with fall risk [26], was higher in the fracture group at 4 weeks after DRF and did not significantly change after 6 months. With the development of wearable sensors, spatiotemporal gait characteristics and their variability in daily life have been increasingly identified. However, few studies have explored the long-term changes in gait characteristics related to falls and fractures. As daily-life gait is influenced by various factors, such as environmental and psychiatric factors [27], changes in patients with DRF could reflect changes in their lifestyle. Conversely, the risk of subsequent fractures is the highest immediately after the initial fracture [28]. Although no subsequent fractures occurred in this study, further research is warranted to determine the relationship between gait changes and subsequent fractures. The in-shoe IMU sensor must play an important role in further evaluating this relationship.

The dorsiflexion angle in the fracture group remained lower, whereas other parameters in the fracture group improved during 6 months. As for vertebral fractures, which are typical fragility fractures as well as DRF, patients with symptomatic vertebral fractures walked with shorter and wider strides at the time of injury. Although those with vertebral fractures show improvement in stride time and stride length over time, even reaching healthy levels again, their gait pattern and stability persist for 6 months, implying a greater risk of incident disability among these patients [29]. Since gait speed is reported to affect other gait parameters, the persistent lower dorsiflexion angle in the fracture group, despite the improvement in gait speed, might be a characteristic of patients with DRF, indicating that patients cannot fully return to healthy states. The dorsiflexion angle in this study, which means the angle between the ground and the sole of the foot, may depend on the movement of all lower extremities. Kyphosis and flexed hip or knee joints, which are common in older people, are related to foot movement or strength [30]. Although further research is needed to determine the cause of this decrease in dorsiflexion angle, the angle could result in stumbling and falls. Older adults with DRF can be assessed as having a high risk of functional decline, particularly those who have access to a health care facility at an early stage. They should receive appropriate intervention to prevent future falls or fractures along with treatment for the initial fracture. Considering that several previous reports have assessed fall risk using machine learning based on gait data from fallers [31,32], our results could be effective in creating a more precise machine learning model for evaluating the risk of falls. Further research is warranted to explore not only the cost of developing sensors and apps but also intervention methods and the extent of fall reduction achievable. Nonetheless, our findings using in-shoe IMU sensors outside the hospital could be valuable for future screening tools to evaluate the risk of falls and fractures.

As for physical assessments, the HGS in the fracture group significantly improved during 6 months after DRF, which is consistent with a previous report [13]. The increased use of the nonaffected hand with DRF in daily life may improve HGS; however, most studies on DRF have focused on the HGS of the affected side, and little is known about that of the nonaffected side. Generally, HGS is associated with health status, including death, falls, and muscle strength [33]. The improvement in HGS could reflect the improvement in health status, and the lower HGS after 6 months of DRF could be associated with a lower degree of health status in the fracture group. Contrastingly, there were no differences in the TUG test results, contrary to our previous reports [13,18]. The average TUG test time in both the fracture and control groups in this study was faster compared to previous studies, which may mean that the TUG test, which involves a few steps and seconds in the laboratory, may not reflect the true physical characteristics. Therefore, daily-life gait analysis for a certain period is needed to identify the slight difference between fracture group patients and healthy controls. By using this in-shoe sensor for a certain period, the lower dorsiflexion angle in patients with DRF was revealed, which remained 6 months after the fracture. We would like to further investigate long-term changes in the gait of these patients.

### Limitations

This study had some limitations. First, the number of participants was small, which could have affected the power of this study. However, individual changes over 6 months appeared to confirm the statistical outcomes and might not have affected the overall conclusions of our study. Second, we observed progress up to 6 months after the fractures. The HGS on the affected side continued to improve beyond 1 year. Long-term changes in more patients with DRF should be further explored. Third, the participants were all Japanese, who had the habit of taking off their shoes indoors. Considering that 6 patients with DRF fell inside their houses without shoes, the results may not accurately reflect the daily free-living assessments of barefoot individuals.

### Conclusions

In summary, we performed a case-control study to investigate the long-term changes in HGS and daily-life gait after DRF. Using an in-shoe IMU sensor, we revealed the recovery of spatiotemporal gait characteristics 6 months after DRF surgery without the participants’ awareness. The dorsiflexion angle in the swing phase and HGS were still lower in the fracture group after 6 months, which could be associated with fracture risk. This in-shoe IMU sensor could be useful for evaluating the future fall and fracture risk outside the hospital and for constructing a new health care system related to preventive medicine using wearable devices outside the hospital.

## Abbreviations

CV: coefficient of variation
DRF: distal radius fracture
HGS: hand grip strength
IMU: inertial measurement unit
TUG: Timed Up and Go
